# The complete chloroplast genome sequence of the medicinal plant *Paeonia delavayi* Franchet. (Paeoniaceae)

**DOI:** 10.1080/23802359.2019.1677528

**Published:** 2019-10-15

**Authors:** Aien Tao, Feiya Zhao, Conglong Xia

**Affiliations:** a College of Pharmacy and Chemistry, Dali University, Dali, China;; b School of Medicine, Tourism and Culture College of Yunnan University, Lijiang, China;; c Key Laboratory, Yunnan Provincial Higher Education Institutions for Development of Yunnan Daodi Medicinal Materials Resources, Yunnan, China

**Keywords:** *Paeonia delavayi*, chloroplast, Illumina sequencing, phylogeny

## Abstract

*Paeonia delavayi* is a medicinal plant commonly used in southwest of China. In this study, we sequenced the complete chloroplast (cp) genome sequence of *P. delavayi* to investigate its phylogenetic relationship in the family Paeoniaceae. The chloroplast genome of *P. delavayi* was 152,872 bp in length with 38.4% overall GC content, including a large single copy (LSC) region of 84,523 bp, a small single copy (SSC) region of 17,053 bp and a pair of inverted repeats (IRs) of 25,646 bp. The cp genome contained 105 genes, including 75 protein coding genes, 27 tRNA genes, and 4 rRNA genes. The phylogenetic analysis indicated *P. delavayi* was closely related to *P. ludlowii*.

*Paeonia* is a very complex genus of the Paeoniaceae family, which includes 35 species in the world (Sang et al. 1997). Most of them are widespread in the northern hemisphere: eastern Asia, central Asia, the Himalayas, the Mediterranean region, and Pacific North America (Stern 1946; Tzanoudakis 1983; Hong et al. 2001). There are 15 species in China (Hong et al. 2001). Plants of this genus have been widely used in traditional Chinese medicine for thousands of years (Jiangsu New Medical College 1977). Among these species, *P. delavayi* is the unique species of China and widely distributed in southwest China which have been used in local medicine in the treatment of rheumatoid arthritis, systemic lupus erythematosus, hepatitis, dysmenorrhoea, muscle cramping and spasms, and fever (Jiangsu New Medical College 1977). However, up to now for such medicinal plant, many studies have mainly focussed on describing its interspecific relationship based on DNA fragments in the genus *Paeonia* (ITS and *matK*) (Chou & Tang 2017) and quantitative analysis of high performance liquid chromatography methods (Hua et al. 2017), the genome information of *P. delavayi* is too little published in GenBank, so that insufficient comprehensive genomic resource is conducted for it. At present, we report the chloroplast genome sequence of *P. delavayi* and find its internal relationships within the family Paeoniaceae, which can provide basic data for further research of genus *Paeonia* species useful genome resource in China.

Fresh and clean leave materials of *P. delavayi* were collected from Yulong county, Yunnan, China (N27°02′36.14″, E100°11′30.33″), and the plant materials and a voucher specimen (No. TAE01) were deposited at Tourism and Culture College of Yunnan University (Lijiang). Total genomic DNA was extracted using the improved CTAB method (Doyle 1987; Yang et al. 2014), and sequenced with Illumina Hiseq 2500 (Novogene, Tianjin, China) platform with pair-end (2 × 300 bp) library. About 8.39 Gb of raw reads with 10,500,370 paired-end reads were obtained from high-throughput sequencing. The raw data was filtered using Trimmomatic v.0.32 with default settings (Bolger et al. 2014). Then paired-end reads of clean data were assembled into circular contigs using GetOrganelle.py (Jin et al. 2018) with *Paeonia rockii* (No. MF488719) as reference. Finally, the cpDNA was annotated by the Dual Organellar Genome Annotator (DOGMA; http://dogma.ccbb.utexas.edu/) (Wyman et al. 2004) and tRNAscan-SE (Lowe and Chan 2016) with manual adjustment using Geneious v. 7.1.3 (Kearse et al. 2012).

The circular genome map was generated with OGDRAW v.1.3.1 (Greiner et al. 2019). Then the annotated chloroplast genome was submitted to the GenBank under the accession number MH463100. The total length of the chloroplast genome was 152, 872 bp, with 38.4% overall GC content. With typical quadripartite structure, a pair of inverted repeats (IRs) of 25,646 bp was separated by a small single copy (SSC) region of 17,053 bp and a large single copy (LSC) region of 84,523 bp. The cp genome contained 105 genes, including 75 protein coding genes, 27 tRNA genes, and 4 rRNA genes. Of these, 18 genes were duplicated in the inverted repeat regions, 9 protein-coding genes, and 5 tRNA genes contain one intron, while three genes (*ycf3*, *rps12* and *clpP*) have two introns.

To investigate its taxonomic status, a total of 11 cp genome sequences of Paeoniaceae species were downloaded from the NCBI database used for phylogenetic analysis. After using MAFFT V.7.149 for aligning (Katoh and Standley 2013), a neighbor-joining (NJ) tree was constructed in MEGA v.7.0.26 (Kumar et al. 2016) with 1000 bootstrap replicates and four Ranunculaceae species (*Coptis chinensis*: MK569483, *Aconitum carmichaelii*: KY407560, *Ranunculus occidentalis*: KX557270, and Anemone raddeana: MK569472) were used as outgroups. The results showed that *P. delavayi* was closely related to *P. ludlowii.* (Figure 1). Meanwhile, the phylogenetic relationship in Paeoniaceae was consistent with previous studies and this will be useful data for developing markers for further studies.

**Figure 1.**
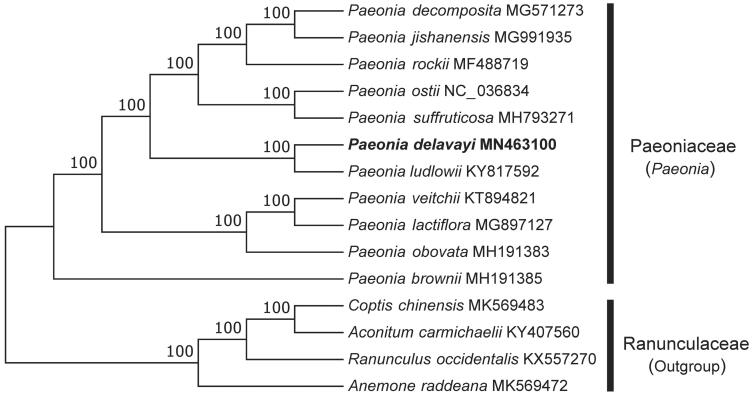
Neighbor-joining (NJ) tree of 11 species within the family Paeoniaceae based on the plastomes using four Ranunculaceae species as outgroups.

## References

[CIT0001] Bolger AM, Lohse M, Usadel B 2014 Trimmomatic: a flexible trimmer for Illumina sequence data. Bioinform. 30(15):2114–2120. 10.1093/bioinformatics/btu170 PMC4103590 24695404

[CIT0002] Chou HH, Tang H 2017 Interspecific relationship among the Wild Species of *Paeonia* Sect. *Moutan* DC with ITS and *matK* Sequence. Bull Bot Res. 37(4):603–612.

[CIT0003] Doyle J 1987 A rapid DNA isolation procedure for small quantities of fresh leaf tissue. Phytochem Bull. 19:11–15.

[CIT0004] Greiner S, Lehwark P, Bock R 2019 OrganellarGenomeDRAW (OGDRAW) version 1.3.1: expanded toolkit for the graphical visualization of organellar genomes. Nucleic Acids Res. 47(W1):W59–W64. 30949694 10.1093/nar/gkz238 PMC6602502

[CIT0005] Hong DY, Pan KY, Nicholas JT 2001 Paeoniaceae In: Wu Z.Y & Raven PH (Eds.). Flora of China. 6th ed. Beijing (China): Science Press; p. 127–132.

[CIT0006] Hua M, Yuan XL, Yang W, Chen J, Hu YL, Tan R, et al. 2017 Analysis of anthocyanins and flavonols in six different colors of petals of *Paeonia delavayi* by high performance liquid chromatography. J West China Forestry Sci. 46(6):40–45.

[CIT0007] Jiangsu New Medical College 1977 Dictionary of Chinese materia medical (in Chinese). Shanghai (China): Science and Technology Press of Shanghai.

[CIT0008] Jin JJ, Yu WB, Yang JB, Song Y, Yi TS, Li DZ 2018 GetOrganelle: a simple and fast pipeline for de novo assembly of a complete circular chloroplast genome using genome skimming data. bioRxiv. 1–11. DOI: 10.1101/256479.

[CIT0009] Katoh K, Standley D 2013 MAFFT multiple sequence alignment software version improvements in performance and usability. Mol Biol Evol. 30(4):772–780. 23329690 10.1093/molbev/mst010 PMC3603318

[CIT0010] Kearse M, Moir R, Wilson A, Stones-Havas S, Cheung M, Sturrock S, Buxton S, Cooper A, Markowitz S, Duran C, et al. 2012 Geneious basic: an integrated and extendable desktop software platform for the organization and analysis of sequence data. Bioinformatics. 28(12):1647–1649. 22543367 10.1093/bioinformatics/bts199 PMC3371832

[CIT0011] Kumar S, Stecher G, Tamura K 2016 MEGA7: Molecular evolutionary genetics analysis version 7.0 for bigger datasets. Mol Biol Evol. 33(7):1870–1874. 27004904 10.1093/molbev/msw054 PMC8210823

[CIT9899395] Lowe TM, Chan PP. 2016 tRNAscan-SE On-line: integrating search and context for analysis of transfer RNA genes. Nucl Acids Res. 44:W54–W57. 27174935 10.1093/nar/gkw413 PMC4987944

[CIT0013] Sang T, Crawford DJ, Stuessy TF 1997 Chloroplast DNA phylogeny, reticulate evolution, and biogeography of *Paeonia* (Paeoniaceae). Am J Bot. 84(8):1120–1136. 21708667

[CIT0014] Stern FC 1946 A study of the genus *Paeonia*. London (UK): Royal Horticultural Society.

[CIT0015] Tzanoudakis D 1983 Karyotypes of four wild *Paeonia* species from Greece. Nord J Bot. 3(3):307–318.

[CIT0016] Wyman SK, Jansen RK, Boore JL 2004 Automatic annotation of organellar genomes with DOGMA. Bioinformatics. 20(17):3252–3255. 15180927 10.1093/bioinformatics/bth352

[CIT0017] Yang JB, Li DZ, Li HT 2014 Highly effective sequencing whole chloroplast genomes of angiosperms by nine novel universal primer pairs. Mol Ecol Resour. 14:1024–1031. 24620934 10.1111/1755-0998.12251

